# 

**Published:** 1871-11

**Authors:** 


### THE STATE BOARD OF DENTAL EXAMINERS.

The annual meeting of this Board, will be held on Wednesday, December 6th, at 12 o'clock M., at the American Hotel, in Columbus. It is desirable that all who design meeting that Board, should be promptly present at that time, that the work may be despatched as speedily as practicable. T.

### CONTENTS.

COMMUNICATIONS.

### The Dental Register

### OF THE WEST.

*Issued Monthly, at $3 a Year in Advance.*

DEVOTED TO THE INTERESTS OF THE DENTAL PROFESSION.

EDITED BY J. TAFT AND G. WATT.

The volume commences in January and closes in December.

The Register being issued monthly is always fresh, therefore indispensable to the practitioner who desires to keep posted up with the new inventions and improvements which are daily developed. Neither expense nor effort will be spared to give value and variety to its contents. Articles will be illustrated with well executed engravings whenever they will add to the interest or assist in explanation.

Its extensive circulation and frequency of issue makes it one of the **BEST DENTAL ADVERTISING MEDIUMS** in the United States.

RATES OF ADVERTISING.

One page, 1 year, $50. Half page, 1 year, $30. Quarter page, or card, 1 year $20.

All advertising bills payable in advance, or at furthest after the issue of the first number containing the advertisement. All transient advertisements to be paid for in advance.

☞ CONTRIBUTIONS SOLICITED.

Address, J. TAFT, or "DENTAL REGISTER," Cincinnati Ohio.

Business Communications, to

**J. TAFT, Publisher,**

**No. 117 West Fourth St.**

